# Traditional cardiovascular risk factors in a cohort of juvenile-onset systemic lupus erythematosus (J-SLE)

**DOI:** 10.1186/1546-0096-9-S1-P261

**Published:** 2011-09-14

**Authors:** Mariana Rodrigues, Luzia Sampaio, Cláudia Moura, Patrícia Costa, Iva Brito

**Affiliations:** 1 Pediatric Rheumatology Unit, Hospital São João, Porto, Portugal; 2 Pediatric Cardiology Department; Hospital São João, Porto, Portugal; 3 Oporto Faculty of Medicine, Portugal

## Background

Despite improved survival in SLE patients, the risk for cardiovascular (CV) death has not diminished. Women under 40 years of age with SLE have a risk of myocardial infarction up to 50-fold greater than controls.

Traditional cardiovascular risk factors are more prevalent among SLE patients than in the general population.

## Aim

To evaluate the prevalence of traditional cardiovascular risk factors in a cohort of adolescents with J-SLE.

## Methods

Cross-sectional and retrospective evaluation of cardiovascular risk factors in a group of J-SLE patients.

## Results

14 J-SLE female patients were included, with disease onset between 6 and 16 years of age (mean 11,9 years), and mean disease duration of 6,5 years (2-15 years).

Five patients had systemic hypertension (4 of them with renal disease), none smoked. 3 patients were overweight (1 with dysplidemia), 11 had normal weight.

Total cholesterol>200 mg/dL in 3 patients, low-density lipoprotein cholesterol>130 mg/dL in 3 patients, triglycerides>150 mg/dL in 2 patients, high-density lipoprotein cholesterol<40 mg/dL in 5 patients. None had fasting glucose > 100 mg/dL.

Statins were used in 2 patients, angiotensin converting enzyme inhibitors in 5 and aspirin in 4. Non-pharmacologic risk factor management was advised in all cases.

There were no overt clinical manifestations of atherosclerotic disease. 2 patients presented with thrombosis (both with antiphospholipid antibodies).

## Conclusion

Given their lifelong exposure to atherogenic risk factors, children and adolescents with SLE are at particularly high risk for premature atherosclerosis and are ideal candidates for primary prevention.

While studies are underway to define optimal strategies and search for risk predictors, the authors would like to emphasize that the early detection of modifiable risk factors can have an important impact on the cardiovascular risk of these patients.

